# Assessment of pulmonary vascular anatomy: comparing augmented reality by holograms *versus* standard CT images/reconstructions using surgical findings as reference standard

**DOI:** 10.1186/s41747-024-00458-w

**Published:** 2024-05-10

**Authors:** Francesco Petrella, Stefania Maria Rita Rizzo, Cristiano Rampinelli, Monica Casiraghi, Vincenzo Bagnardi, Samuele Frassoni, Silvia Pozzi, Omar Pappalardo, Gabriella Pravettoni, Lorenzo Spaggiari

**Affiliations:** 1 Department of Thoracic Surgery, IRCCS European Institute of Oncology, Via Ripamonti 435, 20141 Milan, Italy; 2 Department of Oncology and Hemato-oncology, University of Milan, Via Festa del Perdono 7, 20122 Milan, Italy; 3 Department of Thoracic Surgery, Fondazione IRCCS San Gerardo dei Tintori, Via G. B. Pergolesi, 33, 20900 Monza, Italy; 4 Clinic of Radiology, Imaging Institute of Southern Switzerland (IIMSI), Ente Ospedaliero Cantonale (EOC) Via Tesserete 46, 6900 Lugano, Switzerland; 5 Facoltà di Scienze biomediche, Università della Svizzera italiana (USI), Via Buffi 13, 6900 Lugano, Switzerland; 6 Division of Radiology, IRCCS European Institute of Oncology, Via Ripamonti 435, 20141 Milan, Italy; 7 Department of Statistics and Quantitative Methods, University of Milano-Bicocca, 20126 Milan, Italy; 8 Artiness srl, Viale Cassala 57, 20143 Milan, Italy

**Keywords:** Augmented reality, Holograms, Lung neoplasms, Tomography (x-ray computed), Thoracic surgery

## Abstract

**Background:**

We compared computed tomography (CT) images and holograms (HG) to assess the number of arteries of the lung lobes undergoing lobectomy and assessed easiness in interpretation by radiologists and thoracic surgeons with both techniques.

**Methods:**

Patients scheduled for lobectomy for lung cancer were prospectively included and underwent CT for staging. A patient-specific three-dimensional model was generated and visualized in an augmented reality setting. One radiologist and one thoracic surgeon evaluated CT images and holograms to count lobar arteries, having as reference standard the number of arteries recorded at surgery. The easiness of vessel identification was graded according to a Likert scale. Wilcoxon signed-rank test and *κ* statistics were used.

**Results:**

Fifty-two patients were prospectively included. The two doctors detected the same number of arteries in 44/52 images (85%) and in 51/52 holograms (98%). The mean difference between the number of artery branches detected by surgery and CT images was 0.31 ± 0.98, whereas it was 0.09 ± 0.37 between surgery and HGs (*p* = 0.433). In particular, the mean difference in the number of arteries detected in the upper lobes was 0.67 ± 1.08 between surgery and CT images and 0.17 ± 0.46 between surgery and holograms (*p* = 0.029). Both radiologist and surgeon showed a higher agreement for holograms (*κ* = 0.99) than for CT (*κ* = 0.81) and found holograms easier to evaluate than CTs (*p* < 0.001).

**Conclusions:**

Augmented reality by holograms is an effective tool for preoperative vascular anatomy assessment of lungs, especially when evaluating the upper lobes, more prone to anatomical variations.

**Trial registration:**

ClinicalTrials.gov, NCT04227444

**Relevance statement:**

Preoperative evaluation of the lung lobe arteries through augmented reality may help the thoracic surgeons to carefully plan a lobectomy, thus contributing to optimize patients’ outcomes.

**Key points:**

• Preoperative assessment of the lung arteries may help surgical planning.

• Lung artery detection by augmented reality was more accurate than that by CT images, particularly for the upper lobes.

• The assessment of the lung arterial vessels was easier by using holograms than CT images.

**Graphical Abstract:**

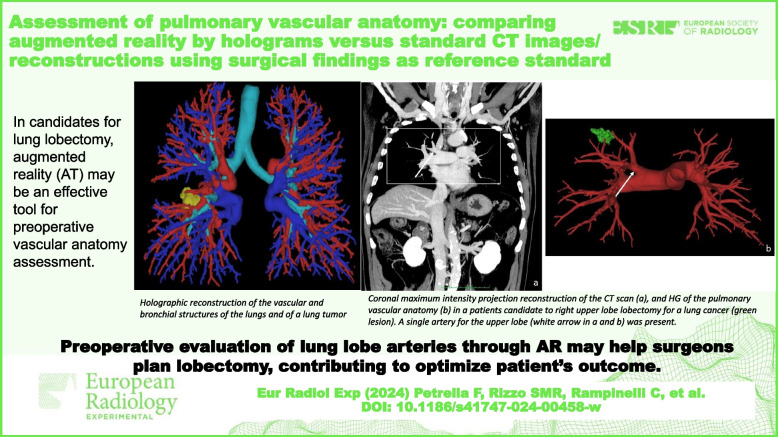

**Supplementary Information:**

The online version contains supplementary material available at 10.1186/s41747-024-00458-w.

## Background

In thoracic surgery, careful preoperative planning, focusing on the pulmonary vascular anatomy of the lobe candidate to lobectomy, is essential in order to reduce the risk of vascular injury, to increase the self-confidence of the operator, to reduce the duration of the procedure, and to optimize the outcome of the procedure [1]. In fact, a 20–30% variation rate from standard vascular anatomy has been reported, thus making lobar resection difficult [2, 3]. This can be even more important when anatomical resection is performed by minimally invasive techniques (video-assisted or robotic-assisted) which currently represent the most common approaches to early-stage lung cancer resections and require careful knowledge of pulmonary vascular anatomy [4]. A proper preoperative assessment of pulmonary artery branch number, location, take-off, and relation with closer anatomical structures results in safer, faster, and overall better vascular dissection during the procedure [5]. Although lobar vein dissection and closure is also an essential step of lung lobectomy, its relevant lower variation rate in number and course usually makes this step of the operation less demanding and more comfortable for the operator [6].

Computed tomography (CT) is a standard of care in the preoperative assessment of lung cancer, not only in terms of oncologic staging but also for pulmonary anatomy evaluation before planning resection [7]. In recent years, augmented reality, including holography, has emerged as a possible further tool for medical imaging with potentially excellent applications in thoracic surgical procedures. The development of holograms (HG) in the 1960s is due to the research of Gabor et al. [8] and was then helped in its growth by many others when light sources became available with the invention of the laser [9, 10]. The enhancement of the real world by using computer-generated data is currently defined as augmented reality (AR) [11]. Thanks to constant advancements obtained by head-mounted displays, the use of holograms (HGs) and AR in clinical medicine and in medical education has become more easily accessible, frequent, and successful.

The primary objective of this prospective study was to compare the performance of standard preoperative CT images and HGs to assess the number of arteries of a lung lobe undergoing resection for curative resection of primary lung cancer. The secondary objective was to compare the perceived easiness in data interpretation by radiologists and thoracic surgeons by using standard CT images and HG.

## Methods

This single-center prospective cohort study was conducted at the European Institute of Oncology (Milan, IT). The local Institutional Review Board approved this study (IRB R1033/19-IEO1088). Written informed consent was obtained from each patient. This trial was registered at ClinicalTrials.gov—Identifier: NCT04227444. Enrollment started on March 1, 2020, and continued to reach the number of patients suggested by the sample size calculation.

### Patient selection

The inclusion criteria were planned anatomical lobar resection for lung cancer, signed and dated informed consent indicating that the patient has been informed of all pertinent aspects of the study, and willingness and ability to comply with study procedures. The exclusion criteria were age younger than 18 years, intraoperative findings leading the surgeon not to perform standard lobectomy, patients unable to provide informed consent, and CT scan performed without administration of iodinated contrast medium.

### CT protocol

The CT examinations were performed either on a 64-slice Siemens Somatom go.Top (Siemens Healthineers, Forchheim, Germany) or on a 64-slice GE MSTC Optima 660 (General Electric Healthcare, Milwaukee, WI, USA), with the following main acquisition parameters, respectively for the two machines: kV 80–120 and 100–140; mA according to automatic exposure control; pitch of 0.8 and 0.516; tube rotation time 0.33 s and 0.5 s; slice thickness 1–3 mm and 1.25–2.5 mm; and collimation 64 × 0.6 and 64 × 0.625.

All scans extended in a cranio-caudal direction in the portal venous phase, 70–90 s after the intravenous administration of Ultravist 370 (Bayer Healthcare, Berlin, Germany) or Visipaque 320 (GE Healthcare, Milan, IT). Quantity and injection rate were adapted to the weight of the patient and to the CT protocol clinically indicated, respectively. All the CT images were archived in digital format and comprised multiplanar reconstructions (in the sagittal and coronal view), as well as maximum intensity projections.

### Augmented reality (HGs) generation

A prototypal software developed by Artiness srl (Milan, Italy) was used to visualize and process the CT scans, generate patient-specific three-dimensional (3D) models (from acquisitions reconstructed with slice thickness 1.25 mm [kernel lung] or 2.5 mm [kernel standard]), and visualize them in a mixed reality (MxR) setting. The CT images were processed using a non-local means filtering [12] to remove noise.

The desktop component of the prototypal software allowed the generation of the 3D models of the lungs, airways, pulmonary arteries, pulmonary veins, and tumor. The lung models were generated using a semiautomated algorithm based on the computation of their isosurface using a marching cubes algorithm and a set of morphological operations. The isovalue was computed automatically, while a set of points on the large airways needs to be manually selected in order to remove them from the model. The airways, pulmonary arteries, and veins were segmented by selecting a series of points along their axes, setting the minimum and maximum HU thresholds to restrict segmentation and generating a deformable model using a level-set formulation. The number of iterations and a set of parameters controlling the level-set propagation were optimized for tubular and branching structures. The tumor isosurface was identified using a marching cubes algorithm.

**Fig. 1:**
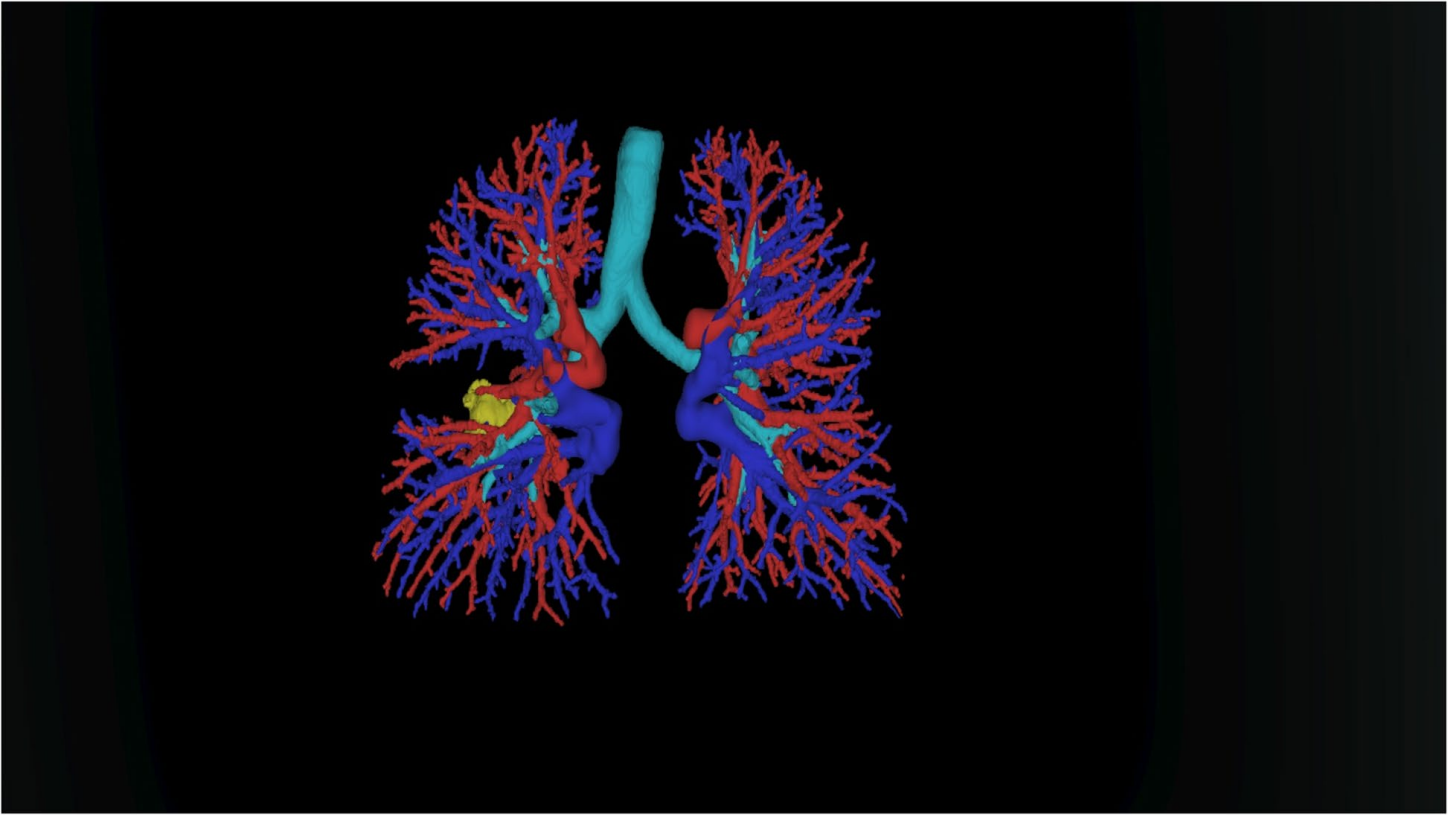
Example of holographic reconstruction of the vascular and bronchial structures of the lungs and of a lung tumor (light blue: bronchial tree; red: arteries; blue: veins; yellow: tumor)

The MxR component allowed the visualization and analysis of the 3D models and the CT images from which they were generated, alongside a set of tools to interact with them. The 3D models can be translated, rotated, scaled, and selectively shown or hidden. An interactive plane could be used to temporarily hide portions of the 3D models and allow an unencumbered visualization of the features of interest. The MxR component runs on a Microsoft HoloLens 2.0 device (Microsoft Corp., Redmond, WA, USA) (Fig. 1; Additional file 1: Video S1)

### Surgery

Patients underwent the planned surgical resection. The operating team reported the exact number of artery branches of the resected lobe. The surgeons who performed the operating procedure were never involved in the evaluation of CT images and HGs, to prevent any source of potential bias.

### Image analysis

One radiologist with 20 years of experience in thoracic CT reading (S.R.) and one thoracic surgeon with 22 years of experience in thoracic surgery (F.P.) analyzed the pseudonymized CT images in one or more of the available planes and reconstructions, until a satisfying evaluation was reached and reported the number of arteries for the lobe resected. In a separate reading session, they analyzed the coded HG by using Microsoft Hololens and reported the number of arteries for the lobe resected. The two reading sessions were performed with a time-lapse of at least 7 days, to ensure a sufficient washout time avoiding comparator review bias. The easiness in artery branch identification on CT images and on HGs was graded by the radiologist and by the thoracic surgeon according to a Likert scale, where identification of the lobar arteries could be “very difficult,” “difficult,” “neutral,” “easy,” or “very easy.”

### Statistical analysis

The sample size in this study was calculated based on the endpoint “number of artery branches of the lobe slated for resection.” The study’s null hypothesis (H_0_) assumed that CTs and HGs would have similar accuracy, meaning both methods would consistently identify the same number of artery branches as observed during surgery. The alternative hypothesis (H_1_) assumed that the accuracy differs between the two methods. For the purpose of sample size calculation, we specifically hypothesized a scenario where HGs would outperform CT images by detecting an average of 0.5 more actual artery branches. Considering a 5% type I error, a sample size of 50 patients was determined to be necessary to achieve an 80% statistical power in refuting H_0_, using a one-sample, two-sided Wilcoxon signed-rank test. Power estimations were based on simulations, with the number of actual artery branches assumed to be distributed according to a zero-truncated Poisson distribution with a mean of 3.6 [1].

Continuous data were reported as median and ranges or interquartile ranges (IQR) or as mean and standard deviation (SD) according to non-normal or normal/near-normal distribution, respectively. Categorical data were reported as counts and percentages.

For the primary analysis, we calculated for each patient *i*:

i. The difference between the number of artery branches identified by the operating team (N_SURG,*i*_) and the mean number of artery branches identified after a CT scan by the radiologist and the surgeon (N_CT,*i*_), as DIFF_CT,*i*_ = N_SURG,*i*_ − N_CT,*i*_ (the greater the difference, the lower the accuracy of the CT)
ii. The difference between the number of artery branches identified by the operating team (N_SURG,*i*_) and the mean number of artery branches identified after HG by the radiologist and the surgeon (N_HG,*i*_), as DIFF_HG,*i*_ = N_SURG,*i*_ − N_HG,*i*_ (the greater the difference, the lower the accuracy of the HG)
iii. The difference between the two above-defined differences, as DIFF_*i*_ = DIFF_CT,*i*_ − DIFF_HG,*i*_ = N_HG,*i*_ − N_CT,*i*_

Wilcoxon’s signed-rank test was used to test the primary null hypothesis. It was also used to test if the difference between CT and HG accuracy in detecting the number of artery branches was the same between the two site groups (upper lobes *versus* middle/lower lobes). If a high agreement was found between the radiologist’s and the surgeon’s evaluation, an average between the two evaluations was considered for further analyses evaluating the accuracy of CTs and HGs in identifying the number of artery branches.

Linear weighted *κ* statistic was used to evaluate the agreement among the number of artery branches detected by the radiologist and the thoracic surgeon.

To evaluate if reading HGs was easier than reading CT, we performed two Wilcoxon signed-rank sum test, the first comparing “CT by radiologist” with “HG by radiologist” evaluations and the second one comparing “CT by surgeon” with “HG by surgeon” evaluations.

*p*-values lower than 0.05 were considered statistically significant.

All analyses were performed with the statistical software SAS 9.4 (SAS Institute, Cary, NC, USA).

## Results

**Fig. 2:**
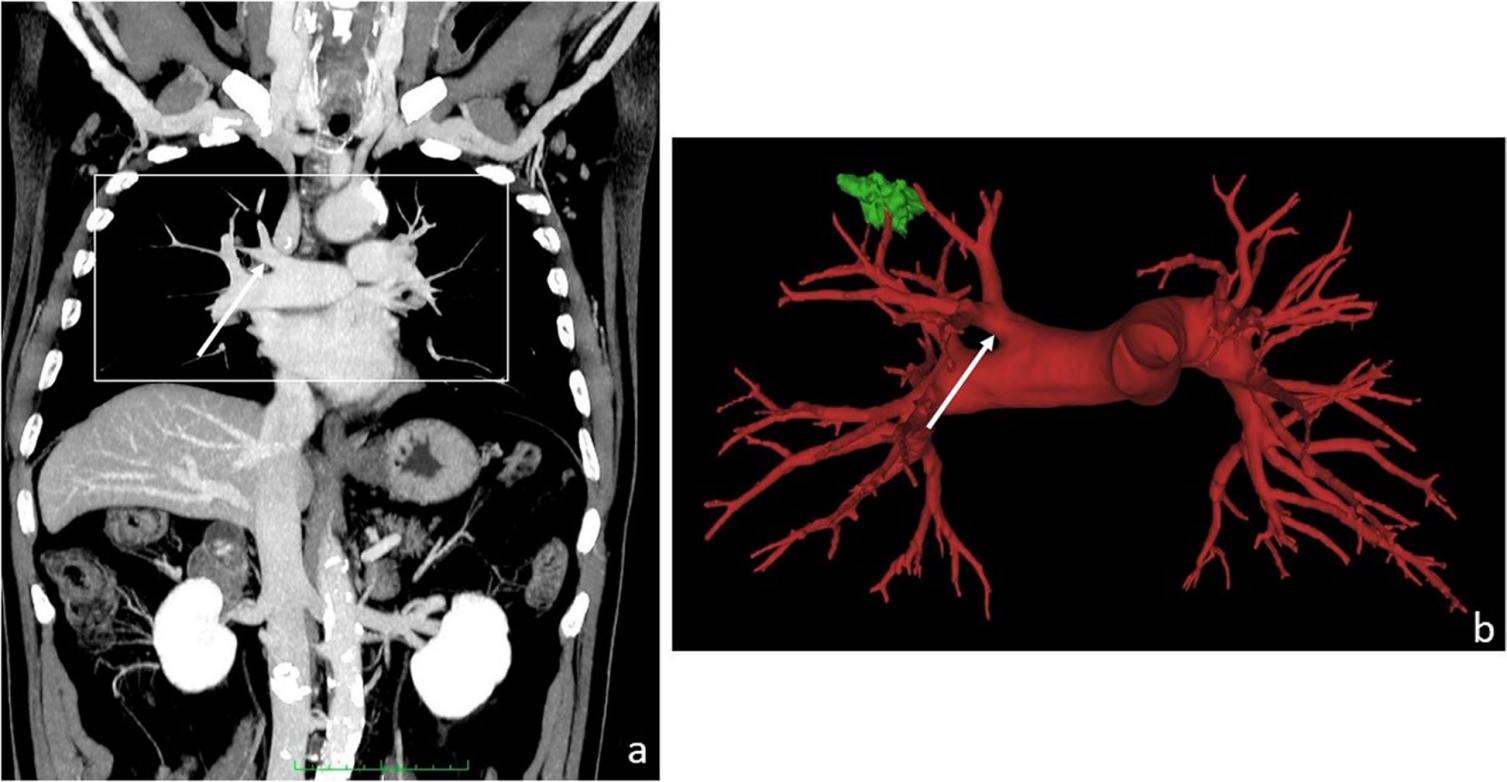
Coronal maximum intensity projection reconstruction of the contrast-enhanced computed tomography scan (**a**), containing a white box indicating where the pulmonary vessels were specifically evaluated, and HG of the pulmonary vascular anatomy (**b**) in a patient candidate to right upper lobe lobectomy for lung cancer (green lesion). The radiologist and surgeon evaluation where concordant with the reference standard (surgery) in indicating a single artery for the upper lobe (white arrow in **a** and **b**)

Enrollment started on March 1, 2020, and ended on March 1, 2022. Sixty-one patients were initially enrolled in this study; six patients were excluded because anatomical lobar resection was not performed because of intraoperative findings; three were excluded because of allergy to iodinated contrast medium, which did not allow generating the HGs. Eventually, 52 patients were included in this study. As shown in Table 1, 28 were females (54%) and 24 were males (46%), with a median age of 67 years (range 38–82); the median number of days between CT scan and surgical procedure was 36 (range 1–113). Right upper lobectomy was performed in 15 of 52 patients (29%), middle lobectomy in 4 patients (8%), right lower lobectomy in 12 patients (23%), left upper lobectomy in 15 patients (29%) (Fig. 2), and left lower lobectomy in 6 patients (12%). Fourteen patients (27%) were operated on by robotic-assisted thoracic surgery, 13 patients (25%) were operated on by video-assisted thoracic surgery, 23 patients (44%) were approached by lateral thoracotomy, and 2 patients (4%) were operated *via* postero-lateral thoracotomy.

**Table 1:** Patient characteristics (*n* = 52)

Variable	Level	Overall (*n* = 52)
Days between CT and surgery, median (min-max)		36 (1–113)
Age at surgery, median (min-max)		67 (38–82)
Sex, *n* (%)	Female	28 (54)
	Male	24 (46)
Site, *n* (%)	Right lower lobe	12 (23)
	Left lower lobe	6 (12)
	Middle lobe	4 (8)
	Right upper lobe	15 (29)
	Left upper lobe	15 (29)
Surgical access, *n* (%)	Robot-assisted surgery	14 (27)
	Lateral thoracotomy	23 (44)
	Posterolateral thoracotomy	2 (4)
	Video-assisted surgery	13 (25)

The radiologist and the thoracic surgeon detected the same number of artery branches of the resected lobe in 44 cases (85%) for CT scans and 51 cases (98%) for HGs. A high agreement was observed for both CT scans (weighted *κ* = 0.81, 95% confidence interval 0.68–0.93), and HGs (weighted *κ* = 0.99, 95% confidence interval 0.96–1.00) (Table 2).

**Table 2:**
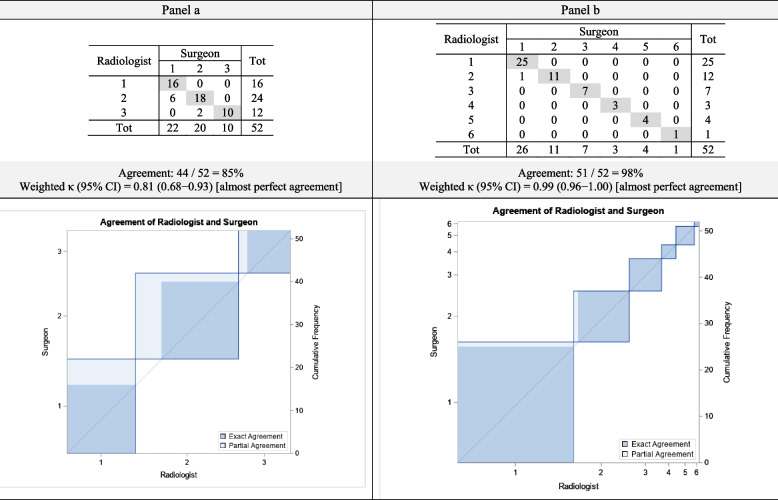
Agreement between radiologist and surgeon evaluations, using computed tomography (panel a) and holograms (panel b)

A high agreement was found between the radiologist’s and the surgeon’s evaluation (Additional file 2: Figs. S1 and S2, Tables S1 and S2).

The mean difference between the number of artery branches detected by surgery (reference standard) and on CT images was 0.31 ± 0.98, whereas it was 0.09 ± 0.37 between surgery and HGs. The *p*-value for the statistical test comparing these two mean differences was 0.433 (Additional file 2: Fig. S3). The mean difference between the number of artery branches detected by surgery and HG was 0.17 ± 0.46 for the upper lobes and -0.02 ± 0.11 for the middle and lower lobes, whereas the mean difference between surgery and CT images was 0.67 ± 1.08 for the upper lobes and -0.18 ± 0.55 for the middle and lower lobes (Additional file 2: Fig. S4).

As shown in Table 3, the difference between CT image and HG accuracy in detecting the number of artery branches in the upper lobes (difference 0.50 ± 1.06) was significantly different from the difference between CT and HG accuracy in the middle and lower lobes (-0.16 ± 0.52) (*p* = 0.024).

**Table 3:** Differences between the number of artery branches detected with surgery (reference standard) and with computed tomography and holograms, divided by site (upper *versus* middle/lower lobes)

Site (lobes)	Comparison	Median (IQR)	Mean ± standard deviation	Min–Max	Difference (mean ± standard deviation)	*p*-value
Upper (*n* = 30)	Surgery *minus* CT	1 (0–1)	0.67 (1.08)	-1–3	0.50 ± 1.06	0.024
	Surgery *minus* HG	0 (0–0)	0.17 (0.46)	-1–1		
Middle/lower (*n* = 22)	Surgery *minus* CT	0 (-1–0)	-0.18 (0.55)	-1–1	-0.16 ± 0.52	
	Surgery *minus* HG	0 (0–0)	-0.02 (0.11)	-1–0		

The* p*-value refers to *H*_0_: the difference between (surgery *minus* CT) and (surgery *minus* HG) is equal among the two groups (upper *versus* middle/lower lobes)

**Fig. 3:**
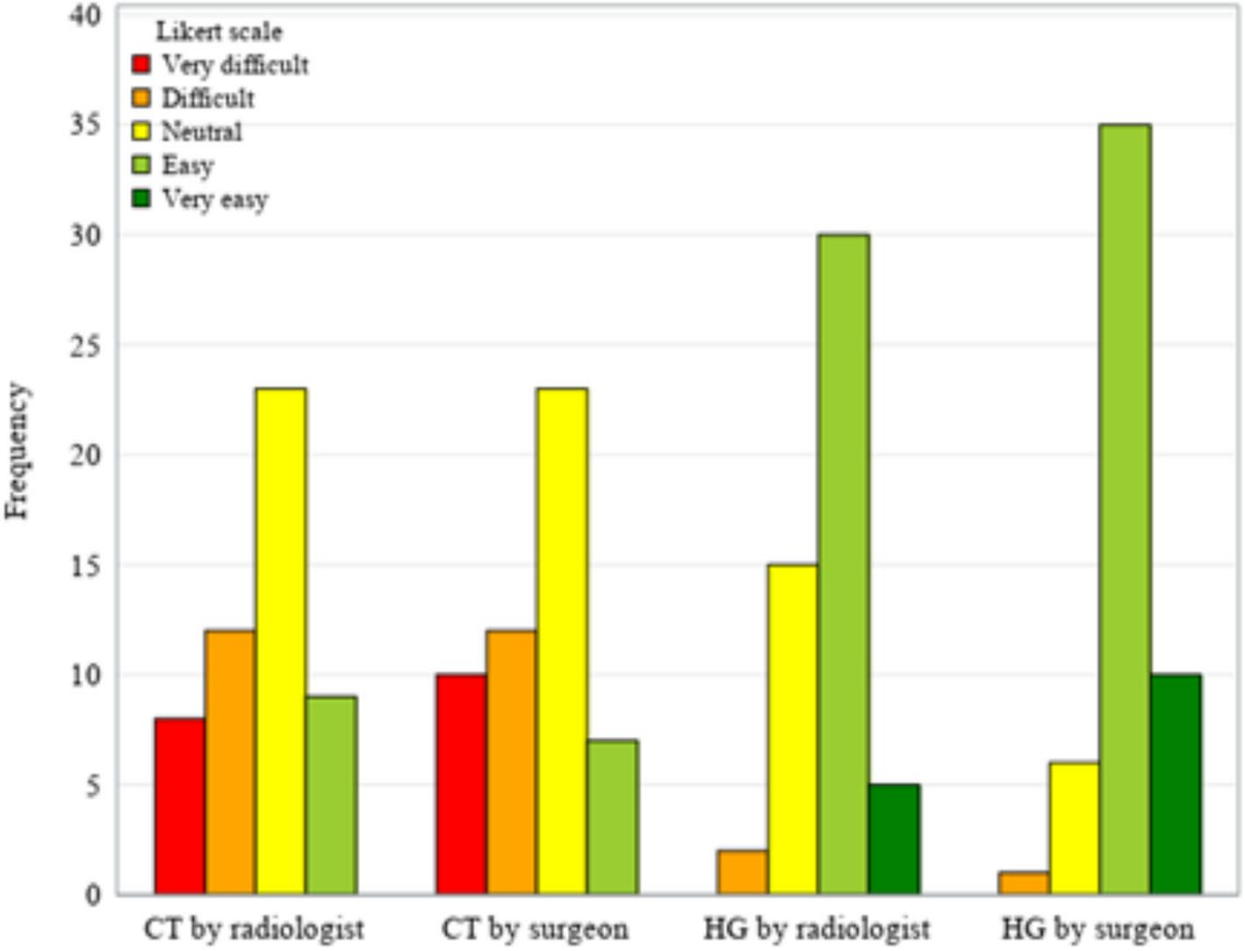
Difficulty/easiness in identifying the arteries: computed tomography (CT) *versus* holograms (HG) (*n* = 52)

**Fig. 4:**
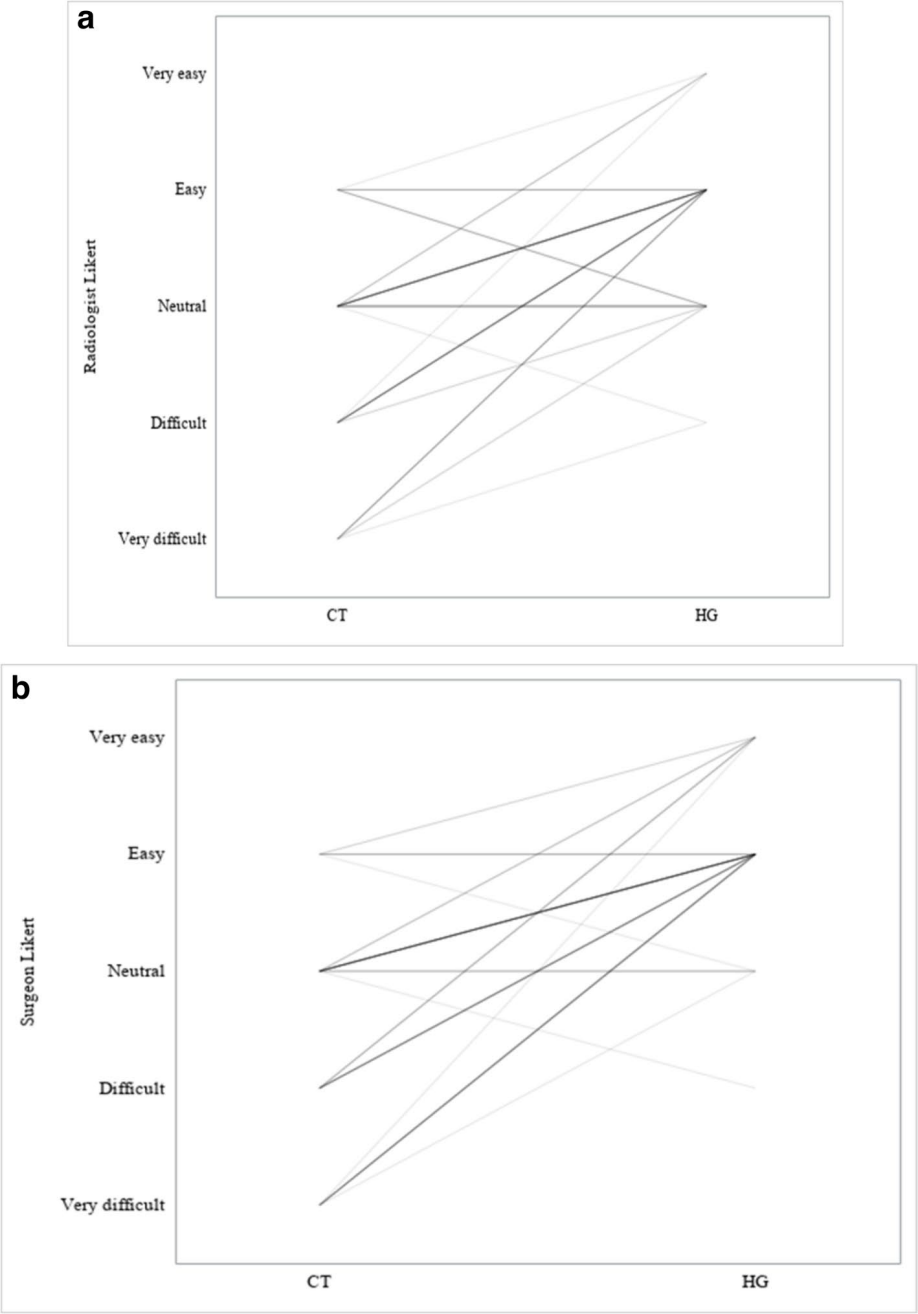
Difficulty in identifying the arteries by radiologist (**a**) and by surgeon (**b**): computed tomography (CT) *versus* holograms (HG) (*n* = 52). Each line represents the paired evaluation of difficulty in identifying the artery branches in a single patient by using CT images or HG. Lines that appear darker and thicker indicate a higher frequency of patients receiving that specific combined evaluation by the radiologist or by the surgeon

Perceived easiness in data interpretation by radiologist and thoracic surgeon using CT images and HGs is reported in Fig. 3, showing that both the radiologist and the thoracic surgeon found it easier to evaluate cases with HG than with CT images. Furthermore, the CT evaluation was sometimes very difficult to read for both clinicians (no very easy cases), whereas the HG evaluation was never very difficult for either clinician, and in some cases, it was very easy. Tables 4 and 5 show data on difficulty in identifying arteries by the radiologist and the surgeon. Figure 4 presents a comparison of the ease with which HGs and CT scans are interpreted by the surgeon (panel a) and the radiologist (panel b), demonstrating a significant advantage (*p* < 0.001) in the simplicity of interpreting HGs over CT scans for both clinicians.

**Table 4:** Difficulty/easiness in identifying the lung arteries by the radiologist: computed tomography (CT) *versus* holograms (*n* = 52)

Radiologist with CT images	Radiologist with holograms					
	Very difficult	Difficult	Neutral	Easy	Very easy	Total
Very difficult	0	1	2	5	0	8
Difficult	0	0	2	9	1	12
Neutral	0	1	7	12	3	23
Easy	0	0	4	4	1	9
Very easy	0	0	0	0	0	0
Total	0	2	15	30	5	52

**Table 5:** Difficulty/easiness in identifying the lung arteries by the thoracic surgeon: computed tomography (CT) *versus* holograms (*n* = 52)

Surgeon with CT images	Surgeon with holograms					
	Very difficult	Difficult	Neutral	Easy	Very easy	Total
Very difficult	0	0	1	8	1	10
Difficult	0	0	0	8	4	12
Neutral	0	1	4	15	3	23
Easy	0	0	1	4	2	7
Very easy	0	0	0	0	0	0
Total	0	1	6	35	10	52

## Discussion

In our prospective cohort, the evaluation of the lung arteries in patients undergoing pulmonary lobectomy was well performed by CT scans and HGs, but HGs outperformed the CT evaluation for upper lobes, where a higher number of vascular variability is present. Furthermore, evaluation of HG is judged to be easier than that of CT, especially for the evaluation done by the surgeon, who is the final user of this data.

Intraoperative dissection, isolation, and closure of artery branches of a lung lobe to be resected represent one of the most challenging steps during standard pulmonary lobectomy. This is mainly due to a high variability rate of vascular pulmonary anatomy and to some anatomical conditions, such as incomplete fissures, bulky tumors, and lymph node distortion of vascular structures which may result in difficult vascular management [2]. In this scenario, a thorough knowledge of the anatomical distribution of pulmonary vessels may help the less expert surgeon to plan the single steps of the surgery well before entering the operating room, and the more expert surgeon to better plan the duration of the surgery. This is even more true in minimally invasive approaches like video-assisted and robot-assisted thoracic surgery, where tactile feedback and manual palpation is not available, although better intraoperative vision might balance this limit [4, 5]. An accurate vascular anatomy knowledge before embarking on pulmonary resection results in a safer, faster, and more confident dissection, reducing potential vascular injuries and optimizing procedure duration and results.

Multidetector CT is the standard imaging technique for preoperative anatomy evaluation, as it allows the evaluation of standard anatomy as well as prognostication in many different settings [13, 14]. In fact, thanks to the progress of the technique in terms of x-ray tubes, detectors, and reconstruction methods, it allows a detailed vision of the anatomy with a short examination time (within a few seconds) and with progressively lower doses of radiation to the patient [15]. The rapidity of the acquisition allows significant motion artifact reduction, while the use of a contrast medium enhances imaging features and resolution [16]. However, the standard axial CT images may be difficult to be translated into a 3D visualization of the surgical field by the operating surgeon. For this reason, postprocessing methods have been developed to create anatomical models closer to the surgical anatomy view. For example, maximum intensity projection is a reconstruction technique increasing the contrast of high attenuation structures, such as lung vessels, maximizing their definition on a two-dimensional plane, while volume rendering is an automatic reconstruction tool based on CT attenuation values of color, brightness, and opacity, generating a three-dimensional image of anatomical structures presenting different densities [17, 18]. In this series, the evaluation of CT images comprised the standard axial acquisitions, the multiplanar reconstructions, and the maximum intensity projections, with no distinction of the contribution given by each one, in order to reproduce a real-world evaluation, where all the series available can be checked and visualized to reach the scope of seeing the arterial branches.

In the last few years, augmented reality has emerged as a promising alternative to standard imaging techniques for preoperative planning in several different districts [18–23]. Spatial recognition, eye tracking, and hand tracking are the most intriguing and encouraging properties of augmented reality. Spatial recognition relies on the ability of full recognition of the world around the operator; eye tracking consists of the capacity to recognize what the operator is seeing, projecting HGs in the visual field of the operator by light rays; hand tracking allows the operator to move, rotate, stretch, touch, and globally interact with the HG by using his/her hands and fingers. Therefore, HGs enhance the clinical scenario by using immersive cinematic photo-real projections, thus providing the operator with a digital overlay of clinical reality [24].

In this study, the use of HGs offered the possibility to interact with a 3D reconstruction of the lungs, where the different anatomical structures could be included or removed according to preference. This allowed us to have a complete and exhaustive view of the lungs and their vessels in one view, which could also be stretched, enlarged, or rotated to look for more precise details. Our findings disclosed that the lobe in which the tumor is located influences HG assessment of the pulmonary arteries to a lesser extent, whereas CT images are less effective than HGs in the assessment of the lung arteries of the upper lobes. According to this result, from a surgical point of view, HGs can significantly contribute to preoperative vascular anatomy assessment, particularly in the case of upper lobectomies that are the most difficult procedures in terms of vascular isolation and dissection.

When the augmented reality technology is available and affordable in the future, we would advise its use in all cases. However, this is still not the case, with costs and time required being the current main disadvantages of the use of this technology. For this reason, given the success in the evaluation of the vessels of the upper lobes, where anatomical variants are more frequent and surgical resection is more challenging, we would advise a thorough evaluation of vascular anatomy of the lung upper lobes before a planned lobectomy by using HGs. This technology may also be of help for teaching less experienced thoracic surgeons before a planned surgery, thus offering a new opportunity for a didactic innovation towards personalized surgery.

Moreover, perceived easiness in data interpretation was higher with HGs for both the radiologist and the thoracic surgeon. In fact, in order to look for vascular details and to follow the individual vascular branches on CT images, it is necessary to scroll up and down the axial images many times, with the intrinsic risk of losing the vessel under evaluation and the need to start again. In this study, HGs proved to be a tool easy to manage, because the offered 3D image is immediate, clear, and easy to interpret, as demonstrated by the evaluation by both the surgeon and the radiologist.

Of note, the HG processing from the standard preoperative CT scan does not need additional radiation dose, different contrast media administration, or redundant procedures for the patients.

This study has some limitations. First, the final sample size (*n* = 52) was relatively small for definite conclusions to be drawn; nonetheless, our preliminary analysis to define the sample size according to the primary endpoint indicated that this would have been a proper sample. Our results are encouraging towards a wider evaluation of the impact of the use of HGs in preoperative planning for candidates for lung resections. Second, the CT acquisitions were performed in the venous phase, as for usual staging at our institution, although we are aware that a CT acquisition in the arterial phase would have resulted in better visualization of the arterial branches in CT images. However, CT for staging of lung cancer usually also includes the abdomen, and in this clinical situation, a single delayed acquisition for thoracic and abdominal contrast-enhanced CT is associated with low contrast-related perivenous artifact and affords better visualization of the lymph nodes, while acceptable vascular and hepatic contrast enhancement is maintained [25]. Since at our institution, the standard CT protocol for lung cancer staging includes only a venous phase of the thorax, we decided to do this first study with possibly nonoptimal images, then given interesting results, we might plan a subsequent study with the use of a modified CT protocol including a single arterial pass on the thorax.

In conclusion, our study suggests that HGs are an effective tool for lung vascular anatomy assessment, showing higher diagnostic consistency across different lobes, when compared to standard preoperative staging CT scans. Its major contribution was observed in the detection of branch arteries of upper lobes, where greater anatomical variability maximizes its benefit. Additionally, the HGs were perceived to be easier to interpret than CT images.

### Supplementary Information

**Additional file 1:** **Video S1.** Supplementary video.

**Additional file 2:** **Fig. S1.** Differences between the number of artery branches detected with surgery (gold standard) and with both computed tomography and holograms by radiologist (Panel A) and by surgeon (Panel B) (*N* = 52). **Fig. S2.** Differences between the number of artery branches detected with surgery (gold standard) and with both computed tomography and holograms by radiologist (Panel A and B) and by surgeon (Panel C and D), among patients with “Upper” (Panel A and C) and “Middle/Lower” (Panel B and D) site. **Fig. S3.** Differences between the number of artery branches detected by surgery (reference standard) and by computed tomography and holograms (*N* = 52). **Fig. S4.** Differences between the number of artery branches detected by surgery (reference standard) and by computed tomography and holograms, between patients with “Upper” (Panel A) and“Middle/Lower” (Panel B) site. **Table S1.** Differences between the number of artery branches detected with surgery (gold standard) and with both computed tomography and holograms by radiologist and by surgeon, divided by site (Upper *versus* Middle/Lower). **Table S2.** Supplementary Table 2. Differences between the number of artery branches detected with surgery (reference standard) and with CT and holograms (HG), by radiologist and by surgeon.

## Data Availability

The datasets used and/or analyzed during the current study are available from the corresponding author upon reasonable request.

## Abbreviations

3D: Three-dimensional
AR: Augmented reality
HG: Hologram
IQR: Interquartile range
MxR: Mixed reality
