# Supplementary material for: Assessment of pulmonary vascular anatomy: comparing augmented reality by holograms versus standard CT images/reconstructions using surgical findings as reference standard
Source: Eur Radiol Exp. 2024 May 10;8:57. doi: 10.1186/s41747-024-00458-w (PMC11082107; doi:10.1186/s41747-024-00458-w)
Supplement: Supplementary file 2 — Additional file 2: Fig. S1. Differences between the number of artery branches detected with surgery (gold standard) and with both computed tomography and holograms by radiologist (Panel A) and by surgeon (Panel B) (N = 52). Fig. S2. Differences between the number of artery branches detected with surgery (gold standard) and with both computed tomography and holograms by radiologist (Panel A and B) and by surgeon (Panel C and D), among patients with “Upper” (Panel A and C) and “Middle/Lower” (Panel B and D) site. Fig. S3. Differences between the number of artery branches detected by surgery (reference standard) and by computed tomography and holograms (N = 52). Fig. S4. Differences between the number of artery branches detected by surgery (reference standard) and by computed tomography and holograms, between patients with “Upper” (Panel A) and“Middle/Lower” (Panel B) site. Table S1. Differences between the number of artery branches detected with surgery (gold standard) and with both computed tomography and holograms by radiologist and by surgeon, divided by site (Upper versus Middle/Lower). Table S2. Supplementary Table 2. Differences between the number of artery branches detected with surgery (reference standard) and with CT and holograms (HG), by radiologist and by surgeon. [file 41747_2024_458_MOESM2_ESM.pdf]

Assessment of pulmonary vascular anatomy: comparing augmented reality by holograms versus standard CT images/reconstructions using surgical findings as reference standard
ELECTRONIC SUPPLEMENTARY MATERIAL

Supplementary Figure 1. Differences between the number of artery branches detected with surgery (gold standard) and with both computed tomography and holograms by radiologist (Panel A) and by surgeon (Panel B) (N=52)

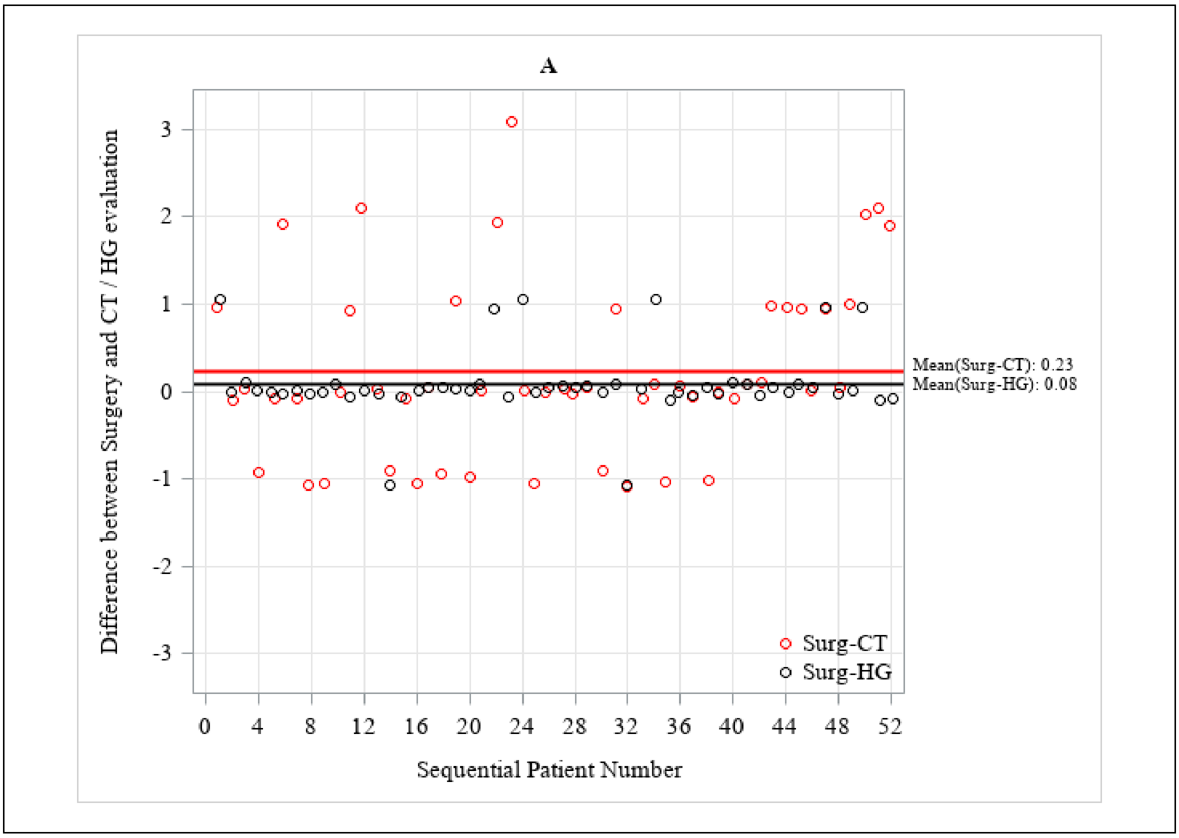

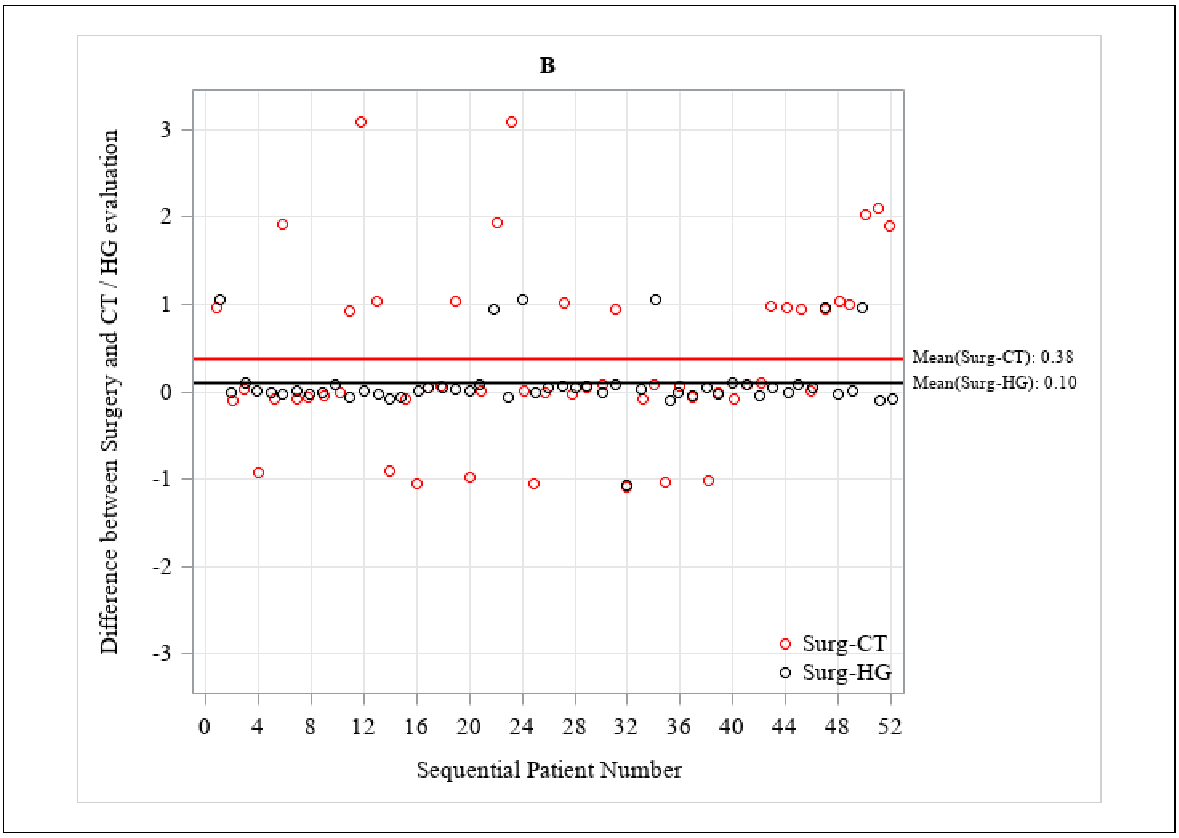
B
Difference between Surgery and CT / HG evaluation
3
2
1
0
-1
-2
-3
Mean(Surg-CT): 0.38
Mean(Surg-HG): 0.10
Surg-CT
Surg-HG
0 4 8 12 16 20 24 28 32 36 40 44 48 52
Sequential Patient Number

Supplementary Figure 2. Differences between the number of artery branches detected with surgery (gold standard) and with both computed tomography and holograms by radiologist (Panel A and B) and by surgeon (Panel C and D), among patients with "Upper" (Panel A and C) and "Middle/Lower" (Panel B and D) site

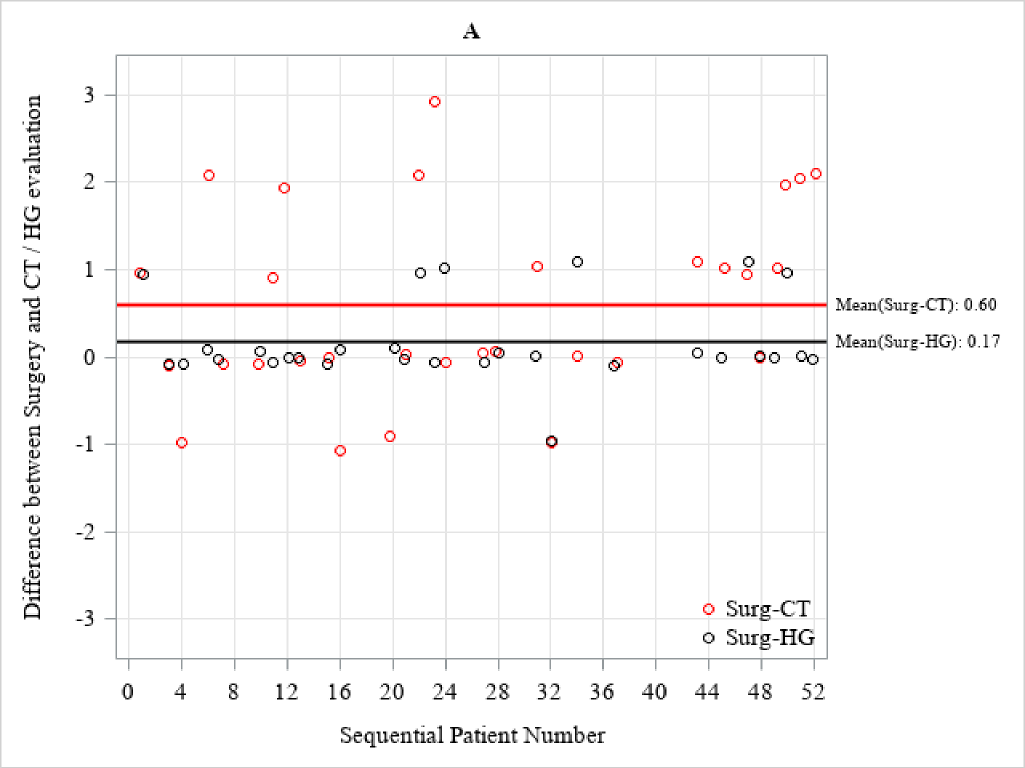

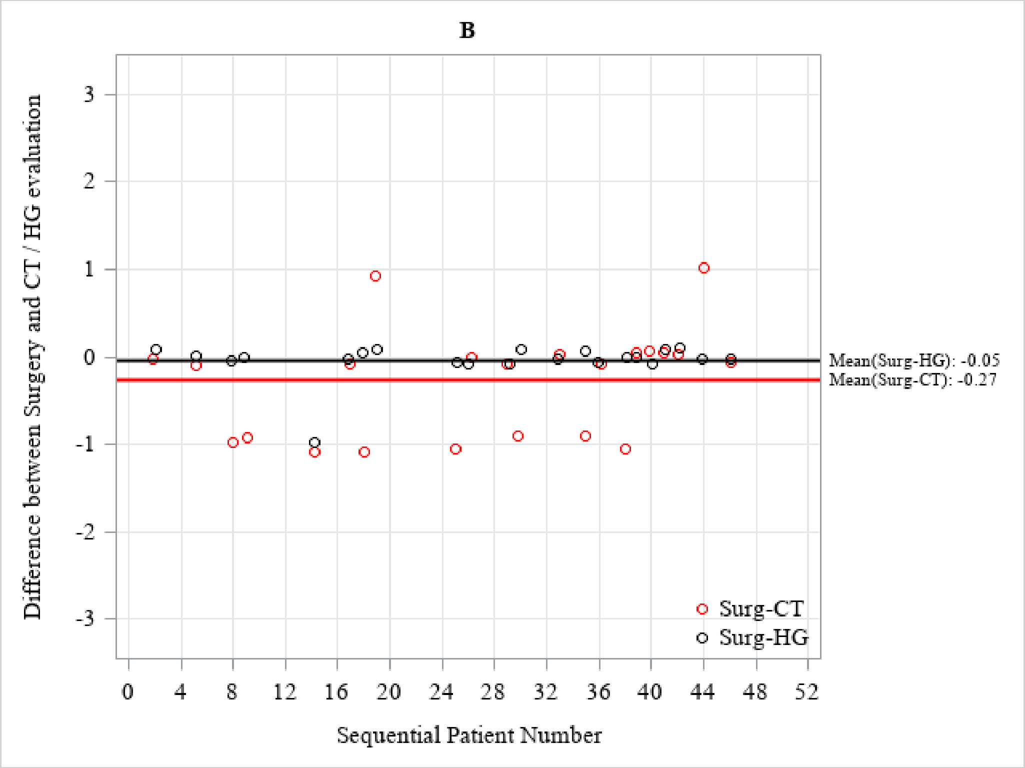

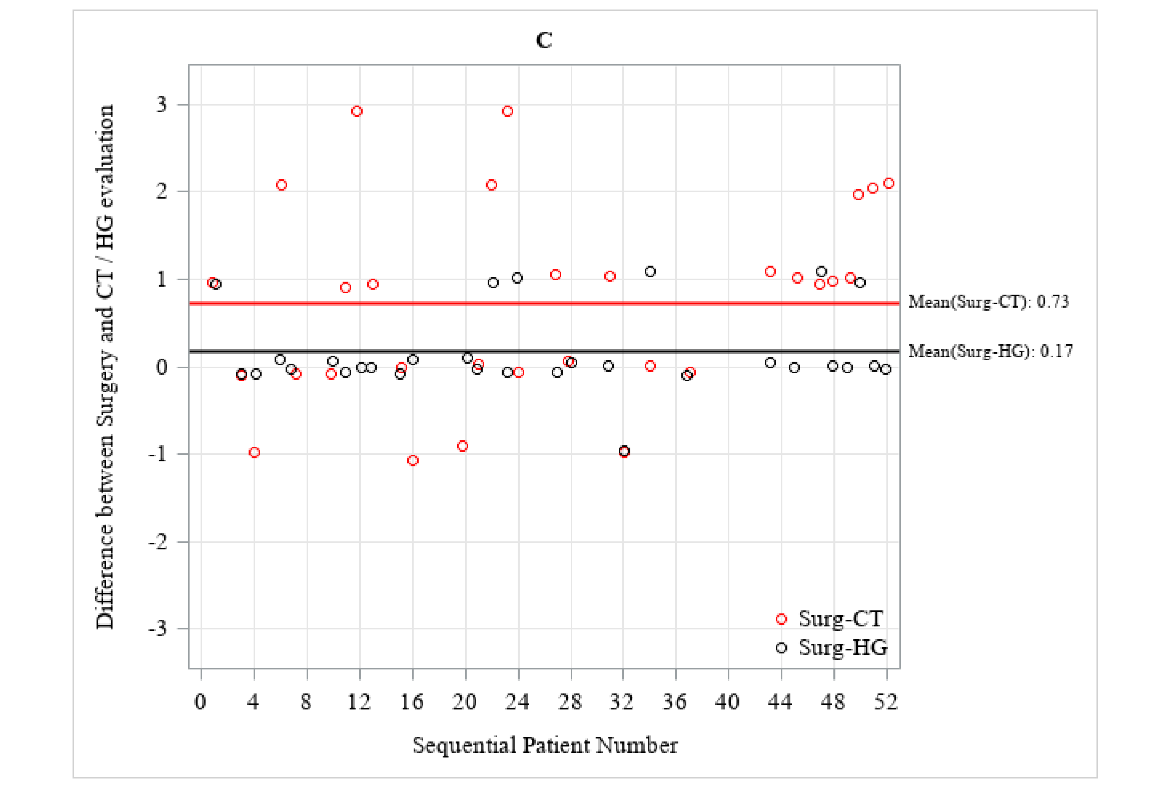
C
Difference between Surgery and CT / HG evaluation
Mean(Surg-CT): 0.73
Mean(Surg-HG): 0.17
Surg-CT
Surg-HG
Sequential Patient Number

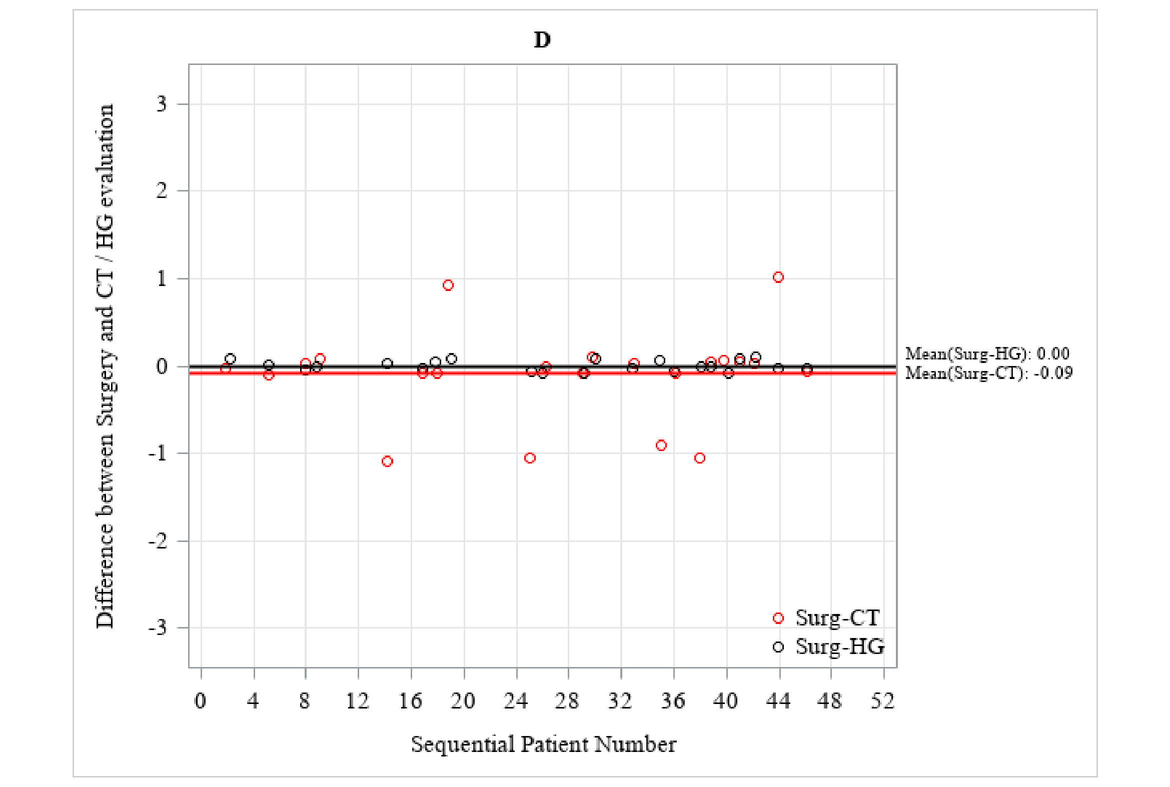
D
Difference between Surgery and CT / HG evaluation
Mean(Surg-HG): 0.00
Mean(Surg-CT): -0.09
Surg-CT
Surg-HG
Sequential Patient Number

Supplementary Figure 3. Differences between the number of artery branches detected by surgery (reference standard) and by computed tomography and holograms (N=52)

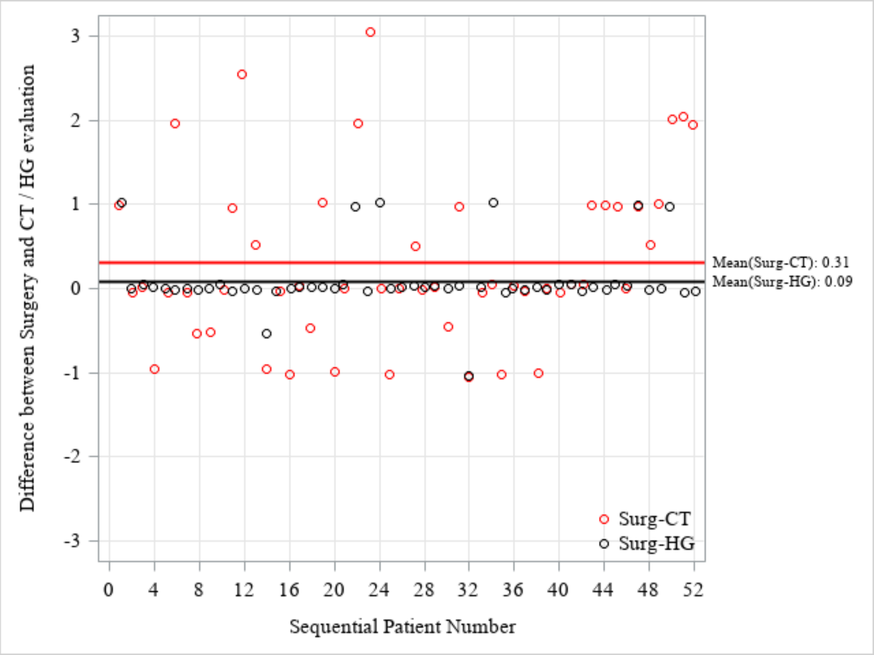

Supplementary Figure 4. Differences between the number of artery branches detected by surgery (reference standard) and by computed tomography and holograms, between patients with "Upper" (Panel A) and "Middle/Lower" (Panel B) site

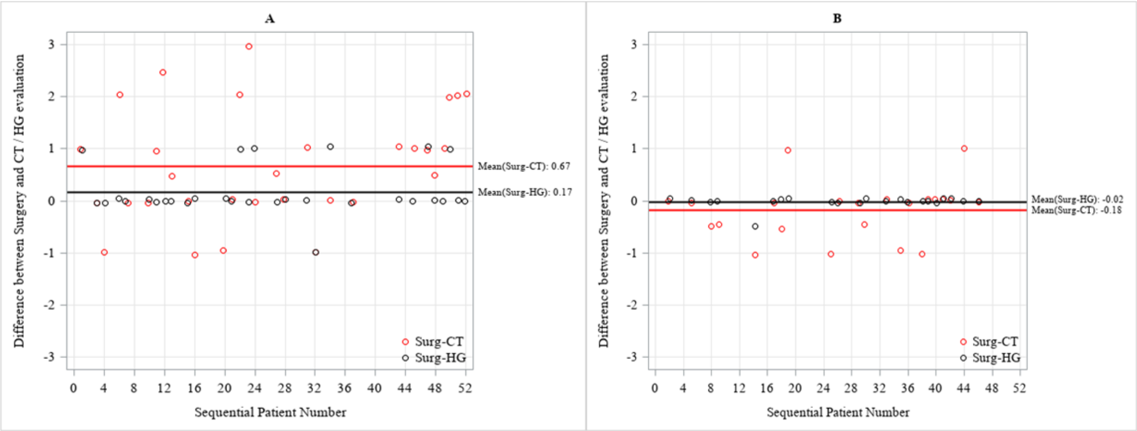

Supplementary Table 1. Differences between the number of artery branches detected with surgery (gold standard) and with both computed tomography and holograms by radiologist and by surgeon, divided by site (Upper vs. Middle/Lower)

N of branches detected	Median (IQR)	Mean (SD)	Min-Max	Mean difference (SD)	P-value
**Surgery - CT by radiologist**	0 (0-1)	0.23 (1.00)	-1 - 3	0.15 (0.94)	0.75
**Surgery - HG by radiologist**	0 (0-0)	0.08 (0.39)	-1 - 1		
**Surgery - CT by surgeon**	0 (0-1)	0.38 (0.99)	-1 - 3	0.29 (0.96)	0.13
**Surgery - HG by surgeon**	0 (0-0)	0.10 (0.36)	-1 - 1		

P-values refers to H0: (Surgery - CT by radiologist) = (Surgery - HG by radiologist) and H0: (Surgery - CT by surgeon) = (Surgery - HG by surgeon)

Supplementary Table 2. Supplementary Table 2. Differences between the number of artery branches detected with surgery (reference standard) and with CT and holograms (HG), by radiologist and by surgeon.

By	Site	N of branches detected	Median (IQR)	Mean (SD)	Min-Max	Mean difference (SD)	P-value
**Radiologist**	**Upper (N=30)**	**Surgery - CT**	0 (0-1)	0.60 (1.07)	-1 - 3	0.43 (1.04)	0.049
		**Surgery - HG**	0 (0-0)	0.17 (0.46)	-1 - 1		
	**Middle/Lower (N=22)**	**Surgery - CT**	0 (-1-0)	-0.27 (0.63)	-1 - 1	-0.23 (0.61)	
		**Surgery - HG**	0 (0-0)	-0.05 (0.21)	-1 - 0		
**Surgeon**	**Upper (N=30)**	**Surgery - CT**	1 (0-1)	0.73 (1.11)	-1 - 3	0.57 (1.10)	0.050
		**Surgery - HG**	0 (0-0)	0.17 (0.46)	-1 - 1		
	**Middle/Lower (N=22)**	**Surgery - CT**	0 (0-0)	-0.09 (0.53)	-1 - 1	-0.09 (0.53)	
		**Surgery - HG**	0 (0-0)	-0.00 (0.00)	0 - 0		

P-value refers to H0: the difference between (Surgery - CT) and (Surgery - HG) is equal among the two groups (Upper vs. Middle/Lower)
